# Effect of aortic regurgitant jet direction on mitral valve leaflet remodeling: a real-time three-dimensional transesophageal echocardiography study

**DOI:** 10.1038/s41598-017-09252-8

**Published:** 2017-08-21

**Authors:** Kensuke Hirasawa, Masaki Izumo, Taro Sasaoka, Takashi Ashikaga, Kengo Suzuki, Tomoo Harada, Mitsuaki Isobe, Yoshihiro J Akashi

**Affiliations:** 1 Department of Cardiovascular Medicine, Tokyo Medical and Dental University, Tokyo, Japan; 2 Division of Cardiology, Department of Internal Medicine, St. Marianna University School of Medicine, Kawasaki, Japan

## Abstract

Chronic aortic regurgitation (AR) induces mitral valve (MV) leaflet enlargement, although, its mechanism still remains unclear. This study aimed to clarify the influence of AR jet directions on the MV apparatus in patients with chronic AR. This study included 69 consecutive patients with severe chronic AR and 17 controls who underwent three-dimensional (3D) transesophageal echocardiography (TEE). The anterior mitral leaflet (AML), posterior mitral leaflet (PML) and MV annulus areas were measured at mid-diastole. All AR patients were classified into the posterior (Group A, n = 38) or non-posterior (Group B, n = 31) group based on the AR jet directions. Both two groups revealed the increased total leaflet areas compared with the controls. No significant differences in the left ventricular volumes, PML or MV annulus area were observed between Group A and B; however, Group A had the larger AML area and greater AML/PML area ratio than Group B (both *P* < 0.01). The multivariate analysis indicated that the posterior AR jet was independently associated with the AML/PML area (*P* < 0.01). 3D TEE depicted geometric differences in the MV apparatus between the different types of AR jet directions. These results may be helpful in understanding the mechanism of MV leaflet remodeling in chronic AR.

## Introduction

Aortic valve diseases, such as aortic regurgitation (AR) and aortic stenosis, have become relatively common in aging developing countries^1, 2^. AR is characterized by the reflux of blood from the aorta into the left ventricle (LV). The overall prevalence of AR detected by color Doppler echocardiography in adults has been reported 4.9% in the Framingham Heart Study^1^ and 10% in the Strong Heart Study^2^. These studies have showed that age is an independent predictor of AR; hence, the increased prevalence of AR is presumed in developed countries^1, 2^. Mitral valve (MV) enlargement in patients with chronic AR has been previously reported^3^. Beaudoin *et al*.^4^ demonstrated that MV leaflet enlargement in patients with chronic AR decreased functional mitral regurgitation (MR) and speculated that LV chamber dilatation was the main cause of MV leaflet growth as an adaptation for MV annulus enlargement^5–7^. In fact, patients with AR are often observed less MR considering their large LV chamber sizes. However, the detailed mechanisms of MV apparatus morphological changes in patients with chronic AR remain unclear. We often observe the asymmetrical growth of the MV in patients with AR, particularly in patients with severe eccentric posterior jets. According to these experiences, we assumed that the MV remodeling in patients with AR should be varied by the directions of AR jets. Today, three-dimensional (3D) transesophageal echocardiography (TEE) can provide a detailed quantification of the MV apparatus, including MV annulus area, anterior mitral leaflet (AML) and posterior mitral leaflet (PML) areas, and MV opening area^8–13^. We hypothesized that the directions of AR jets affect the MV apparatus and trigger AML enlargement in patients with chronic AR. Here, this study aimed to investigate the MV apparatus by using 3D TEE to understand the mechanism of MV morphological changes in patients with chronic AR.

## Methods

### Study subjects

**Figure 1:**
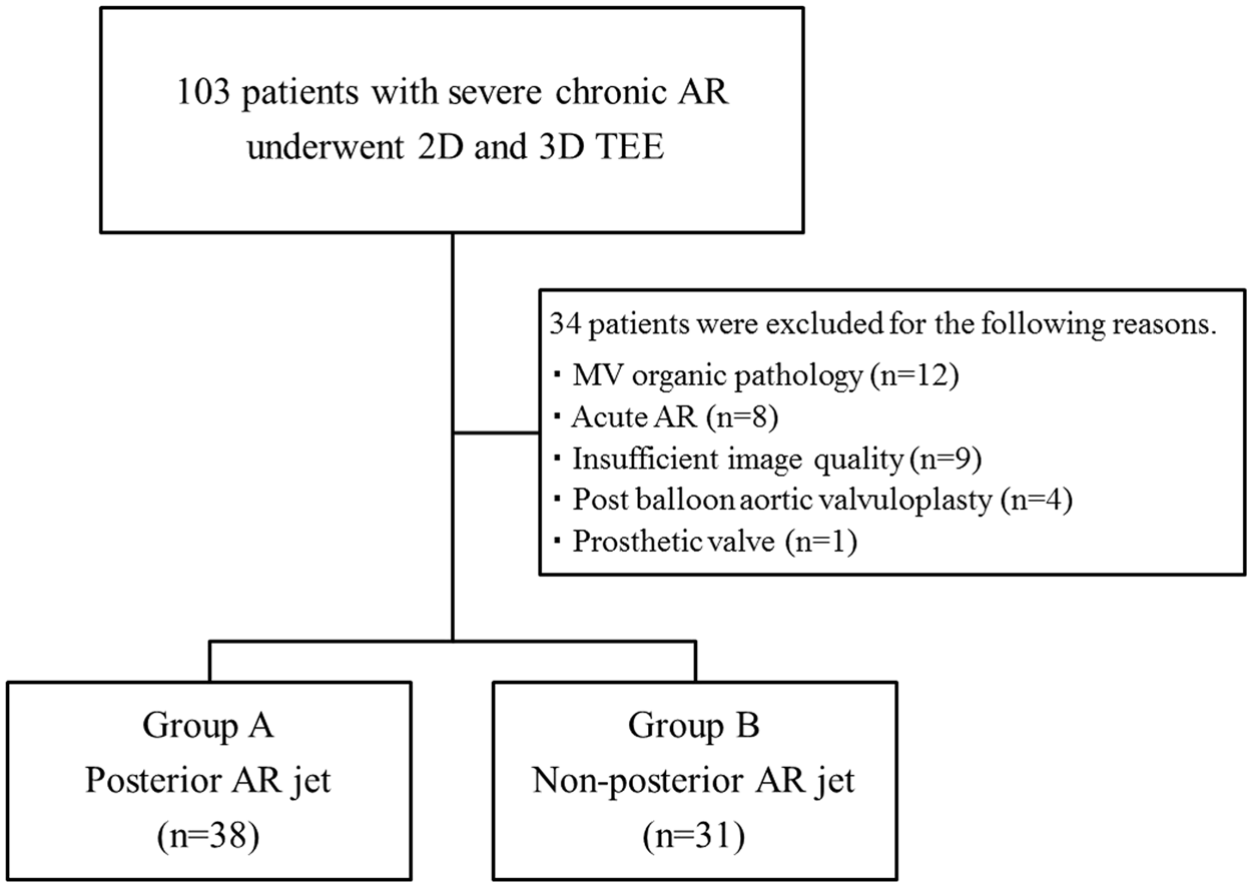
Description of study design. Patients with severe aortic regurgitation (AR) were enrolled and classified based on AR jet direction into the following two groups: posterior jet group and non-posterior jet group.

This study consisted of 103 consecutive patients with severe AR and 17 normal controls who underwent two-dimensional (2D) and 3D TEE between February 2012 and November 2016. The exclusion criteria included insufficient TEE image qualities (n = 9), MV organic pathology (prolapse, severe rheumatic disease, and severe valve calcification; n = 12), acute AR caused by infective endocarditis and aortic root dissection (n = 8), post-balloon aortic valvuloplasty (n = 4), and prosthetic mitral and aortic valves (n = 1). Ultimately, 69 patients and 17 controls were registered (Fig. 1). No patients with Marfan syndrome or any connective tissue diseases were included in this cohort. The controls were defined as 1) the patients who had TEE indications, such as suspected patent foramen ovale, infective endocarditis, and intracardiac thrombus, and 2) those without structural deficiencies, arrhythmia, or other abnormal findings. This study was performed in accordance with the ethical principles set forth in the Declaration of Helsinki; the study protocol was approved by the Institutional Committee of Human Research of St. Marianna University School of Medicine, Kanagawa, Japan. Written informed consent was given from all study patients prior to their enrollment.

### 2D and 3D transesophageal echocardiography (TEE)

**Figure 2:**
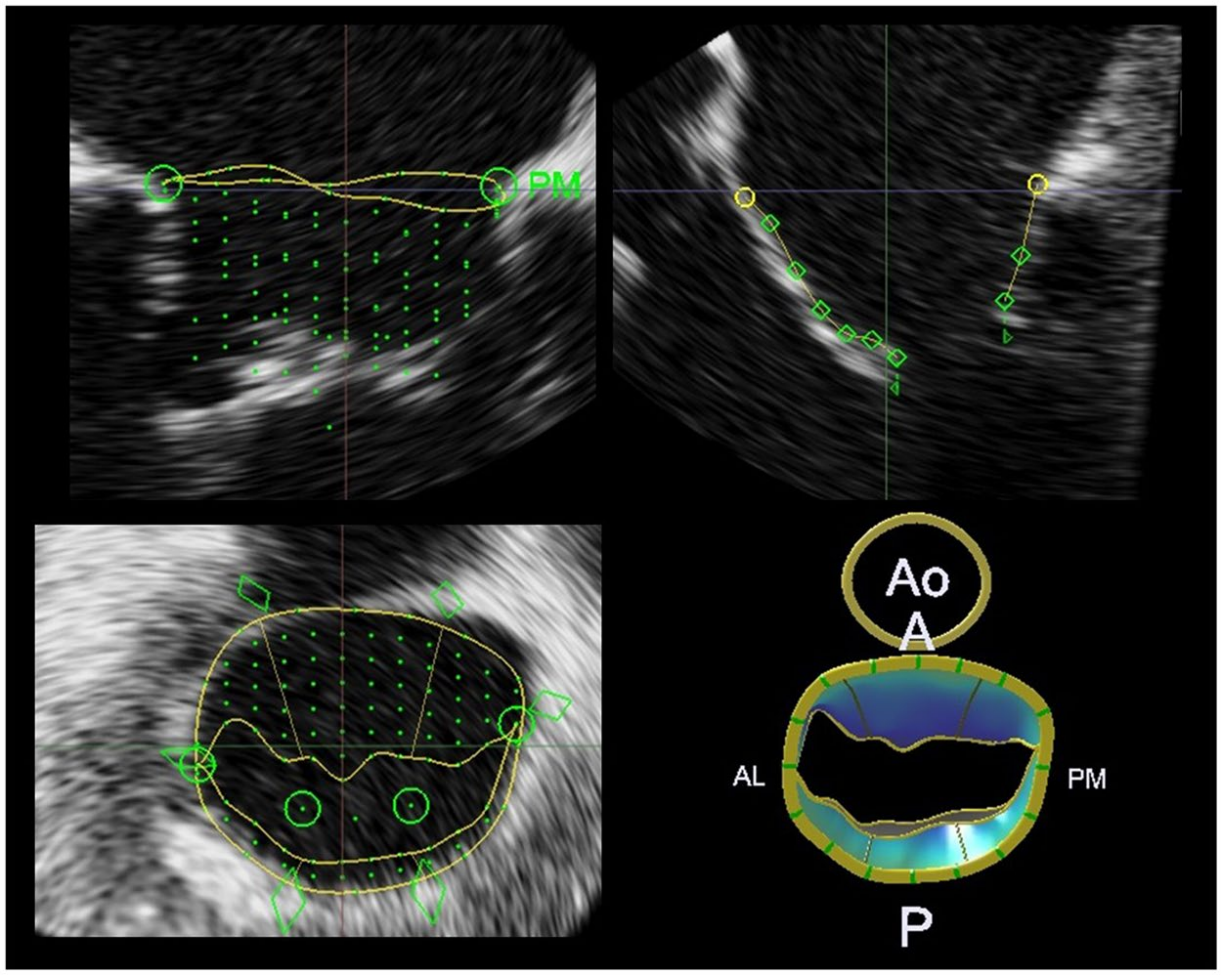
Three-dimensional (3D) mitral valve (MV) morphological analysis in a mid-diastolic frame using MV Navigator ^A.I^. (MVN ^A.I.^). The mitral annulus was manually initialized; then, both the anterior mitral leaflet (AML) and posterior mitral leaflet (PML) were manually traced. The result was displayed as a color-coded model and each parameter was automatically calculated. AL, anterolateral; PM, posteromedial; Ao, aorta; A, anterior; P, posterior.

All patients underwent 2D and 3D TEE using an iE33 or EPIQ7c ultrasound imaging system equipped with an X7–2t transducer (Philips Healthcare, Andover, Massachusetts, USA) displaying both 2D and real-time 3D images. Under topical anesthesia in the pharynx and intravenous sedation with propofol, the transducer was advanced into the esophagus. The directions of AR jets from the mid-esophageal position with a 2D long-axis view (approximately 135°) of the aortic valve and MV were evaluated. The directions of AR jets were visualized using color Doppler 2D TEE and classified into the following two groups, the posterior jet (Group A) or non-posterior jet (Group B) group (Fig. 1). The probe was positioned at the mid-esophageal level for scanning the 3D images. 3D TEE was performed using a fully sampling matrix array transducer (X7–2t). Initially, gain settings were optimized using the narrow-angled acquisition mode which allowed 3D TEE pyramidal volume of approximately 30° × 60°. The zoomed 3D TEE images of the entire MV and gated full volume sets were then acquired in single cardiac cycle. All 3D TEE data were digitally stored for offline analysis (QLAB cardiac 3DQ, Philips Medical Systems, Andover, Massachusetts). The real-time 3D volume data were obtained with the 3D zoom mode which could display a smaller magnified volume image. The 3D data of the MV was analyzed by using QLAB 10.1 and a dedicated software (MV Navigator ^A.I.^ and Cardiac 3D quantification, 3DQ, Philips Healthcare, Andover, Massachusetts, USA) for quantifying MV geometry. The MV leaflets and annulus were traced at mid-diastole and the area of each leaflet and the MV annulus were quantified in all patients (Fig. 2). The mid-diastolic frame was selected and the long-axis view of the MV apparatus was employed to determine the anterior, posterior, anterolateral, and posteromedial annular coordinates. The annulus was manually outlined by defining annular points in each plane rotated around the axis perpendicular to the mitral annular plane. The leaflets were manually traced in the multiple parallel long-axis planes spanning the annulus from commissure to commissure. Subsequently, a color-coded 3D-rendered surface representing a topographical map of the MV leaflets was displayed. The software then automatically generated the measurements of key parameters of annular dimensions and geometry, including each leaflet area. The MV opening area was quantified in the same mid-diastolic frame as previously described using 3DQ. The AML/PML area ratio was then defined to identify asymmetrical remodeling in the patients with posterior AR jets. The AML/MV annulus area ratio was also employed to identify the different types of remodeling in the posterior AR jet group compared with remodeling induced by LV chamber dilatation and MV annulus dilatation.

### TTE

Comprehensive transthoracic echocardiography was performed in all patients using a commercially available ultrasound system within 3 months before or after TEE. The 2D and Doppler images were recorded according to the guidelines described by American Society of Echocardiography (ASE)^14^. The Teichholz formula in B-mode was used to measure an aortic root diameter, left atrial diameter as an anterior-posterior diameter, and LV end-diastolic and end-systolic diameters^15^. LV end-diastolic and end-systolic volumes were measured according to the Simpson’s biplane method. LV ejection fraction (EF) was calculated by the following formula: [(end-diastolic volume – end-systolic volume)/end-diastolic volume] ×100^16^. The peak early and late diastolic velocities of LV inflow (E and A velocity), deceleration time of early diastolic velocity, and peak early diastolic velocity on the septal corner of the mitral annulus (E’) were measured in the apical four-chamber view. The severity of AR was assessed by an integrate approach using TTE according to the ASE guidelines^17, 18^: effective regurgitant orifice area (EROA) > 0.30 cm^2^ by the proximal isovelocity surface area (PISA) method, vena contracta (VC) width > 6 mm, and and/or LV end-diastolic diameter (LVDd) > 55 mm were defined as severe AR. The PISA method successfully measured EROA in 38 patients (55%) and VC width in 58 patients (84%). When these quantifications were not feasible by some reasons, the patients who had visually significant AR with LV dilatation (LVDd > 55 mm) and holo-diastolic reverse flow of the descending aorta were identified as having severe AR.

### Statistical analysis

All continuous variables were presented as mean ± standard deviation; the categorical data were presented as number and percentage. The data for the posterior and non-posterior groups were compared using the Student’s *t*-test, chi-squared test, and Fisher exact test as appropriate. The categorical data were compared with the χ^2^ test and Fisher exact test. *P*-values < 0.05 were considered to indicate significant differences. The univariable and multivariable analyses using single and multiple regression models were performed to assess the influence on the AML and PML areas and the MV opening area/MV annulus area ratio. In the multivariable analysis, the factors possibly affecting MV remodeling were selected (i.e. age, LV end-diastolic volume, body surface area, and posterior AR jets). The intraobserver and interobserver variabilities of the MV annulus area, AML and PML areas, and MV opening area were assessed using an intra-class correlation coefficient for absolute agreement (ICCa) and Bland-Altman methods. Data analyses were performed using JMP^®^ 10 (SAS Institute Inc., Cary, NC, USA).

## Results

### Baseline characteristics

Baseline characteristics are shown in Table 1. The causes of AR were congenital aortic valve disease (n = 14, 21%; 12 patients with bicuspid valves and 2 patients with quadricuspid valves), aortic root dilatation (n = 23, 34%), prolapse (n = 8, 12%), and degenerative changes (n = 23, 34%). Of the 69 patients, 38 patients were classified to Group A and 31 patients to Group B (Fig. 1). No significant differences in age, sex, height, weight, body surface area, or the presence of atrial fibrillation were found between the two groups. The most common reasons for AR were aortic root dilatation in Group A and degenerative changes in Group B (Table 1). TTE characteristics are presented in Table 2. No differences in TTE parameters were observed between the two groups.

**Table 1:** Baseline characteristics.

	Group A:	Group B:	*P* value
	posterior AR jet	non-posterior AR jet	
	(n = 38)	(n = 31)	
Age, years	66 ± 12	64 ± 14	0.52
Males, (%)	30 (79)	22 (71)	0.44
Height, cm	166 ± 10	166 ± 10	0.97
Weight, kg	65 ± 13	62 ± 13	0.40
Body surface area, m^2^	1.72 ± 0.2	1.69 ± 0.2	0.56
Atrial fibrillation, (%)	4 (11)	5 (16)	0.49
AR etiology			
Congenital AV disease, (%)	11 (29)^†^	3 (10)^†^	0.03
Aortic root dilatation, (%)	15 (39)	8 (27)	
Prolapse, (%)	3 (8)	5 (16)	
Degenerative change, (%)	9 (24)^†^	15 (50)^†^	
Medications			
Beta-blocker, (%)	10 (26)	10 (32)	0.53
ACE-inhibitor or ARB, (%)	23 (61)	19 (61)	0.95
Calcium channel blocker, (%)	13 (34)	12 (39)	0.70
Diuretic, (%)	10 (26)	14 (45)	0.10

Values are mean ± SD or n (%).

AR, aortic regurgitation; AV, aortic valve; ACE, angiotensin converting enzyme; ARB, angiotensin II receptor blocker.

†Significant difference.

**Table 2:** Two-dimensional transthoracic echocardiographic characteristics.

	Group A:	Group B:	*P* value
	posterior AR jet	non-posterior AR jet	
	(n = 38)	(n = 31)	
Severity of MR, (%)			0.89
none	5 (13)	3 (10)	
mild	29 (76)	23 (77)	
moderate	4 (11)	5 (17)	
severe	0 (0)	0 (0)	
LVEF, %	54 ± 13	57 ± 11	0.39
AoD, mm	39 ± 5	36 ± 7	0.08
LAD, mm	37 ± 7	40 ± 8	0.11
LV end-diastolic diameter, mm	62 ± 7	58 ± 9	0.08
LV end-systolic diameter, mm	43 ± 9	40 ± 9	0.16
LV end-diastolic volume, ml	189 ± 65	164 ± 62	0.12
LV end-systolic volume, ml	92 ± 50	75 ± 45	0.15
Peak E velocity, m/s	62 ± 20	65 ± 19	0.53
E wave deceleration time, ms	211 ± 59	214 ± 57	0.86
Peak A velocity, m/s^†^	72 ± 19	79 ± 25	0.25
E/A^†^	0.87 ± 0.35	0.95 ± 0.62	0.57
E’, cm/s	6.1 ± 1.9	6.1 ± 2.1	0.99
E/E’ ratio	10 ± 4	11 ± 5	0.31
TRPG, mmHg	27 ± 8	27 ± 10	0.96

Values are mean ± SD or n (%). ^†^Except for patients with atrial fibrillation.

AR, aortic regurgitation; MR, mitral regurgitation; LVEF, left ventricular ejection fraction; AoD, aortic root diameter; LAD, left atrium diameter; LV, left ventricular; TRPG, tricuspid regurgitation pressure gradient.

### MV apparatus

**Figure 3:**
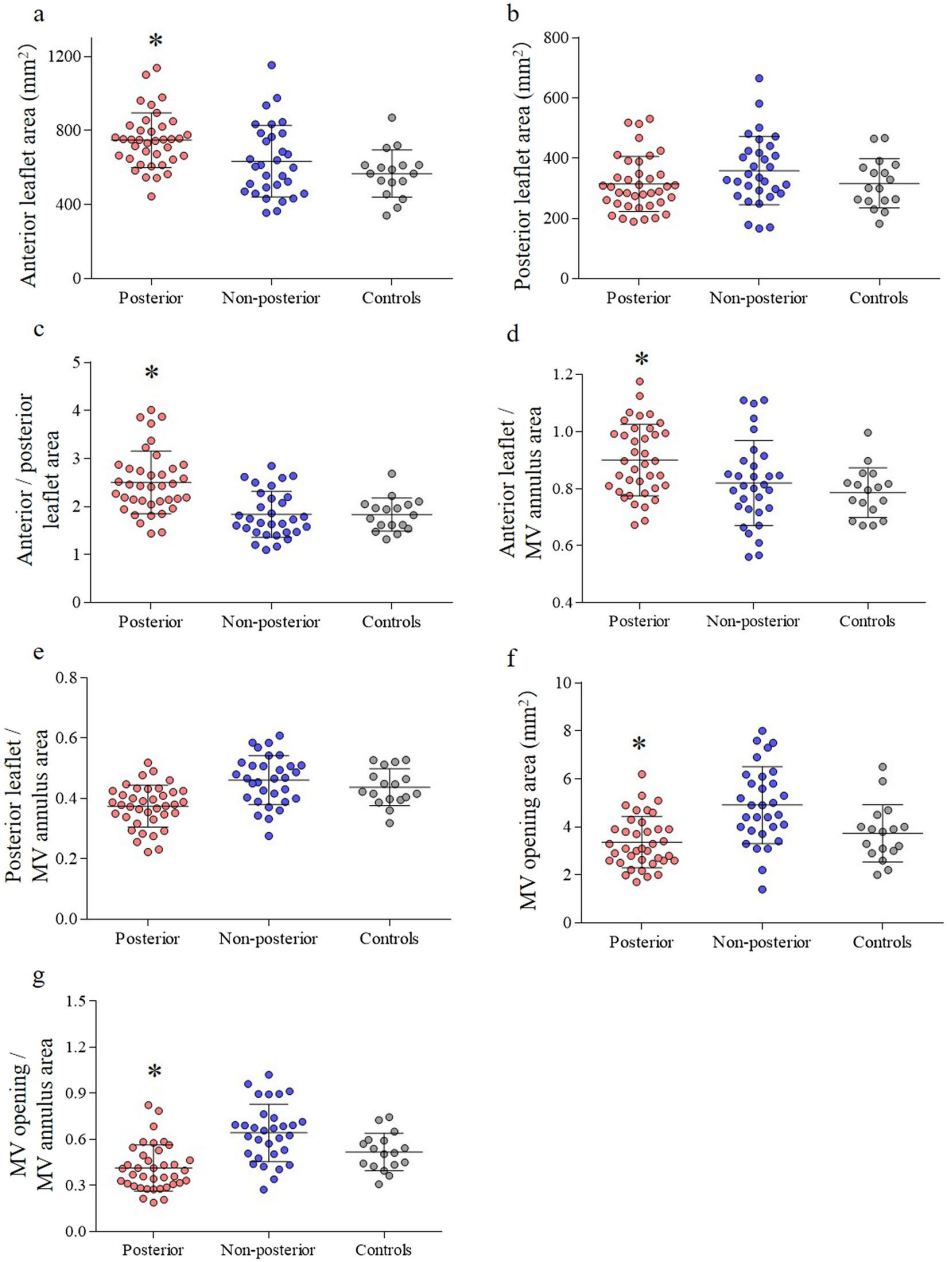
MV geometry measurements in each group and controls. (**a**) AML area (**b**) PML area (**c**) AML area/PML area ratio (**d**) AML area/MV annulus area ratio (**e**) PML area/MV annulus area ratio (**f**) MV opening area. (**g**) MV opening area/MV annulus area. *P < 0.05 compared with other group and controls.

**Figure 4:**
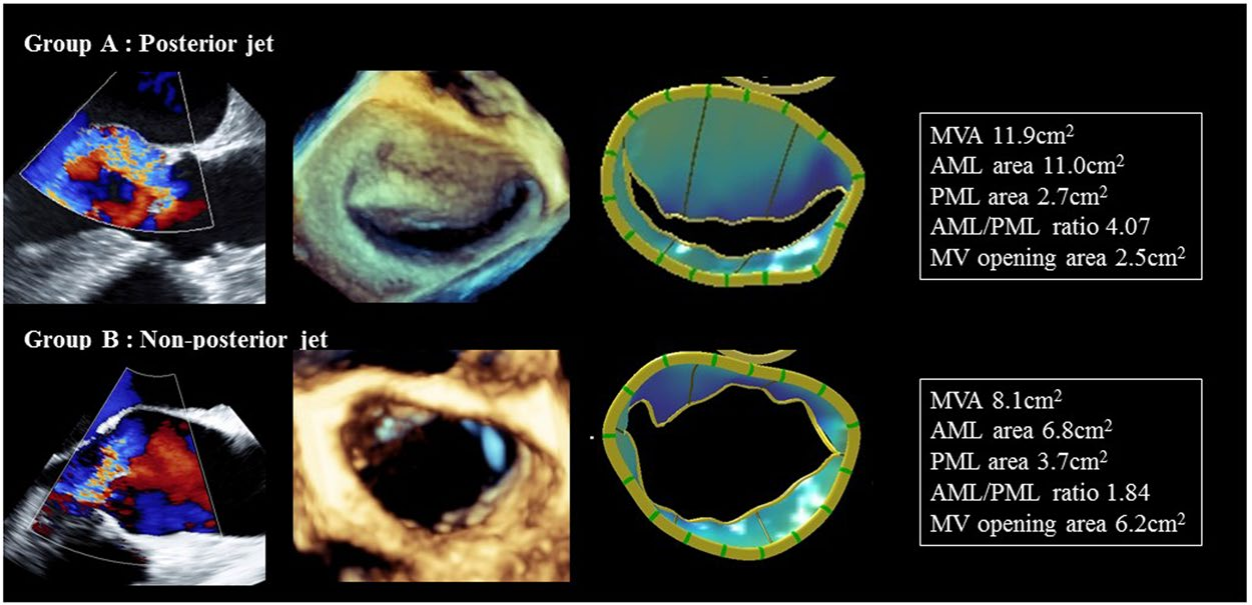
Representative examples of Group A (posterior jet) and Group B (non-posterior jet). MVA, mitral valve annulus; AML, anterior mitral leaflet; PML, posterior mitral leaflet; MV, mitral valve.

The average frame rate of MV 3D images was 14 ± 6 Hz and the average heart rate (HR) was 66 ± 13 bpm. The results of MV analysis using 3D TEE are shown in Table 3 and Fig. 3. No significant differences were observed in the MV annulus area at both mid-systole and mid-diastole between the two groups (*P = *0.06 and *P = *0.13). Both posterior and non-posterior groups revealed the increased total leaflet area compared with the controls (10.6 ± 2.1 vs 9.9 ± 2.8 vs 8.8 ± 1.9 cm^2^). The AML area was significantly larger in Group A than Group B (*P* < 0.01, Fig. 3a); however, the PML area did not significantly differ between the two groups (*P = *0.08, Fig. 3b). The AML and PML areas and the AML area/MV annulus area ratio were significantly greater in Group A because of AML enlargement (*P* < 0.01 and *P* = 0.02; Fig. 3c and d). In contrast, Group A had the smaller PML area/MV annulus area ratio, MV opening area, and MV opening area/MV annulus area ratio at mid-diastole than Group B (all *P* < 0.01; Fig. 3e,f, and g). The representative examples are shown in Fig. 4.

**Table 3:** Mitral apparatus quantification using three-dimensional transesophageal echocardiography.

	Group A:	Group B:	Controls	*P* value (Group A vs. Group B)
	posterior AR jet	non-posterior AR jet		
	(n = 38)	(n = 31)	(n = 17)	
Mid systolic MV annulus area, cm^2^	8.5 ± 1.8^†^	7.7 ± 1.7	7.3 ± 1.6	0.06
Mid diastolic MV annulus area, cm^2^	8.4 ± 1.7^†^	7.7 ± 1.9	7.2 ± 1.5	0.13
Anterior leaflet area, cm^2^	7.5 ± 1.5^†^	6.3 ± 1.9	5.7 ± 1.3	<0.01
Posterior leaflet area, cm^2^	3.1 ± 0.9	3.6 ± 1.1	3.2 ± 0.8	0.08
Mid diastolic MV opening area, cm^2^	3.4 ± 1.1	4.9 ± 1.6^†^	3.7 ± 1.2	<0.01
Anterior/posterior leaflet area ratio	2.5 ± 0.7^†^	1.8 ± 0.5	1.8 ± 0.3	<0.01
Anterior leaflet/MV annulus area ratio	0.9 ± 0.13^†^	0.82 ± 0.15	0.79 ± 0.09	0.02
Posterior leaflet/MV annulus area ratio	0.37 ± 0.07^†^	0.46 ± 0.08	0.44 ± 0.06	<0.01
MV opening area/MV annulus area ratio	0.41 ± 0.15^†^	0.64 ± 0.19^†^	0.52 ± 0.12	<0.01

Values are mean ± SD or n (%). ^†^P < 0.05 vs. controls.

AR, aortic regurgitation; MV, mitral valve.

### Associations between MV geometry and clinical/echocardiographic findings

Table 4 shows the results of single and multiple regression analyses to identify the factors correlating with the AML/PML areas and MV opening area/MV annulus area ratio. In the single regression analysis, the posterior AR jet was significantly correlated with the greater AML/PML area ratio (*P* < 0.001). In the multiple regression analysis, age and posterior AR jet were independently associated with the AML/PML area ratio (*P* < 0.001). Meanwhile, the posterior AR jet was also associated with the MV opening area/MV annulus area ratio (Table 5).

**Table 4:** Univariable and multivariable analyses of anterior mitral leaflet area/posterior mitral leaflet area ratio

	Univariable analysis			Multivariable analysis		
	standardized partial regression coefficient	95% CI	*P* value	standardized partial regression coefficient	95% CI	*P* value
Age, years	−0.089	(−0.015, 0.006)	0.41	−0.204	(−0.022, 0.001)	0.08
Male	0.009	(−0.144, 0.156)	0.94			
Body surface area, cm^2^	−0.087	(−0.928, 0.397)	0.43	−0.209	(−1.435, 0.101)	0.09
LVEF, %	−0.002	(−0.011, 0.011)	0.99			
LVEDV, ml	0.210	(−0.0007, 0.003)	0.22	−0.034	(−0.003, 0.002)	0.78
LVESV, ml	0.027	(−0.002, 0.003)	0.81			
Posterior AR jet	0.501	(0.193, 0.474)	<0.001	0.538	(0.216, 0.500)	<0.001

CI, confidence interval; LVEF, left ventricular ejection fraction; LVEDV, left ventricular end-diastolic volume; LVESV, left ventricular end-systolic volume; AR, aortic regurgitation.

Data are presented from the single and multiple regression analyses.

**Table 5:** Univariable and multivariable analyses of mitral valve opening area/mitral valve annulus area ratio.

	Univariable analysis			Multivariable analysis		
	standardized partial regression coefficient	95% CI	*P* value	standardized partial regression coefficient	95% CI	*P* value
Age, years	−0.104	(−0.005, 0.002)	0.34	−0.062	(−0.004, 0.002)	0.56
Male	−0.090	(−0.063, 0.026)	0.41	−0.090	(−0.072, 0.030)	0.42
Body surface area, cm^2^	−0.042	(−0.236, 0.160)	0.70			
LVEF, %	0.064	(−0.002, 0.004)	0.56			
LVEDV, ml	−0.186	(−0.001, 0.0001)	0.09	−0.108	(−0.001, 0.0004)	0.35
LVESV, ml	−0.149	(−0.001, 0.0003)	0.17			
Posterior AR jet	−0.568	(−0.155, −0.074)	<0.001	−0.534	(−0.150, −0.066)	<0.001

CI, confidence interval; LVEF, left ventricular ejection fraction; LVEDV, left ventricular end-diastolic volume; LVESV, left ventricular end-systolic volume; AR, aortic regurgitation.

Data are presented from the single and multiple regression analyses.

### Associations between significant MR and clinical/echocardiographic findings

Table 6 shows the results of single regression analysis; the total leaflet area and the total leaflet area/MV annulus area ratio were closely associated with moderate and severe MR. (p < 0.05)

**Table 6:** Univariable analysis of significant mitral regurgitation.

	Univariate analysis		
	standardized partial regression coefficient	95% CI	*P* value
Age, years	1.028	0.972–1.028	0.337
Male	2.609	0.690–9.859	0.158
Body surface area, cm^2^	0.528	0.022–12.516	0.693
LVEF, %	0.964	0.917–1.014	0.153
LVEDV, ml	1.005	0.966–1.014	0.255
LVESV, ml	1.009	0.997–1.021	0.138
Posterior AR jet	1.300	0.342–4.944	0.700
MV annulus area, cm^2^	1.003	1.000–1.007	0.059
AML area, cm^2^	0.893	0.794–1.006	0.153
PML area, cm^2^	0.849	0.779–1.003	0.116
Total leaflet area, cm^2^	0.927	0.917–1.000	0.044
Total leaflet/MV annulus area ratio	0.968	0.952–1.000	0.043
AML/PML area ratio	0.405	0.116–1.408	0.155

CI, confidence interval; LVEDV, left ventricular end-diastolic volume; LVESV, left ventricular end-systolic volume; AR, aortic regurgitation.; MV, mitral valve; AML, anterior mitral leaflet; PML, posterior mitral leaflet.

### Reproducibility

The intraobserver variabilities assessed by ICC were 0.96 for MV annulus area (95% confidential interval, CI; 0.90 to 0.99), 0.95 for AML area (95% CI, 0.86 to 0.98), 0.92 for PML area (95% CI, 0.79 to 0.97), and 0.93 for MV opening area (95% CI, 0.82 to 0.97). The interobserver variabilities on these areas were 0.96 (95% CI, 0.90 to 0.99), 0.97 (95% CI, 0.91 to 0.99), 0.95 (95% CI, 0.87 to 0.98), and 0.93 (95% CI, 0.82 to 0.97). The Bland-Altman method showed that interobserver and intraobserver variabilities were −19.9 ± 56.5 and −36.0 ± 54.5 mm^2^ for MV annulus area, −21.2 ± 68.7 and −33.1 ± 53.5 mm^2^ for AML area, −1.2 ± 47.4 and −6.7 ± 35.9 mm^2^ for PML area, and −0.24 ± 0.47 and 0.10 ± 0.48 cm^2^ for MV opening area.

## Discussion

LV chamber remodeling induces mitral leaflet growth^5, 7, 19^. Some studies^4–7^ have already demonstrated that patients with chronic AR, particularly those with lower EF, have a larger total mitral leaflet area and this phenomenon probably reduces MR^4^. Our study population also had the significant correlations between significant MR and MV total leaflet area and total leaflet area/MV annulus area ratio. Although the detailed mechanism of leaflet enlargement in AR patients remains unknown, an adaptation to increased LV chamber and distorted MV annulus shape are considered as possible factors. Some of the earlier conducted studies using 3D transthoracic echocardiography failed to quantify each leaflet area. Nowadays, the MV apparatus depicted by 3D TEE allows software quantifying the MV annulus area, total leaflet area, and AML and PML areas, individually^8–10^. The present study using 3D TEE demonstrated the geometric changes of the MV apparatus in patients with chronic AR according to the directions of AR jets. In our study, the MV total leaflet area was larger in both posterior and non-posterior groups than the controls reported in the earlier studies^4–7^. Furthermore, we found that the posterior AR jets induced AML growth, while PML remained unchanged. These results support the theory that MV remodeling could prevent functional MR in both posterior and non-posterior groups. Although relatively symmetrical remodeling was observed in the patients with non-posterior AR jets, MV remodeling in those with posterior AR jets was asymmetrical. Accordingly, we presumed that the mechanisms of MV remodeling might be different based on the directions of AR jets. AML growth in the patients with posterior AR jets might also be affected by the multiple factors. These speculations were due to mechanical stretch induced by MV annulus dilatation and chronic tethering caused by LV remodeling^4–7^. In fact, the patients with posterior AR jets in our study tended to have greater LV volume and larger MV annulus area. One study has described that LV dilatation did not affect AML length^20^, although, it still remains controversial. In the present study, the patients with posterior AR jets had the larger AML area than those with non-posterior AR jets, whereas the PML areas did not significantly differ among these groups. These results support the prevalence of asymmetrical remodeling of the MV leaflet in chronic AR patients with posterior jets and this phenomenon cannot be solely explained by mechanical stretch of annulus dilatation caused by LV remodeling. Meanwhile, AML enlargement is triggered by mechanical stretch caused by AR jets. Shear stress and mechanical pressure on the heart valves induce the biosynthesis of extracellular matrix materials, such as collagen and proteoglycan, and valvular remodeling^21–25^. The collagen synthesis of the heart valves is also found to be dependent on the degree and duration of mechanical stretch^26^. The posterior jets in patients with chronic AR cause cyclic stress towards the AML over the long period of time. The results of our study thus suggested that the mechanisms of MV remodeling might differ based on the directions of AR jets. Recently, leaflet remodeling has become a focus of attention as one of the important factor to prevent the occurrence of MR^4, 27^; however, the mechanism of leaflet remodeling has not been fully investigated. The earlier studies speculated mechanical stretch induced by MV annulus dilatation and chronic tethering caused by LV remodeling. Our study results additionally demonstrated the other mechanism, AML remodeling triggered by mechanical stretch from the AR jets. The present study also demonstrated the restricted MV opening at mid-diastole in patients with posterior AR jets. Although this finding was suggested for a long time^28^, the quantitative assessment using 3D has not been performed. The influence of MV opening restriction at mid-diastole on the hemodynamics has not been fully elucidated; however, all the trans-mitral flow data, such as peak A velocity, E’, E/E’, and deceleration time, were not significantly different between the AR-jet groups in our study. These findings suggested that MV closing induced by a AR jet during mid-diastole might not affect functional mitral stenosis. These transformations of mitral leaflet area in patients with chronic AR also provide important information to determine treatment policy. Firstly, the changes of the MV apparatus should be considered when assessing AR patients for a MV repair. One study^20^ suggested that AML length should be used as a reference of annular dilatation because AML enlargement in patients with posterior AR jets requires an adequate MV ring size. The other study reported that AML length was a predictor of MV reparability^29^. Thus, the careful assessment should be provided to these patients while discussing MV repair. Secondly, AML enlargement may occasionally cause systolic anterior motion (SAM) of the MV. Maron and his colleagues^30^ demonstrated that an AML length/LV outflow tract (LVOT) diameter was associated with subaortic obstruction in patients with hypertrophic cardiomyopathy. They speculated that the elongated AML leaflet might have the potential for mitral-septal contact and create LV outflow obstruction. Several studies also reported that long AML and a mismatch of AML/PML length were the predictors of SAM and LVOT obstruction in patients with LV hypertrophy^31, 32^. Some reports^33, 34^ have described the possibility of LVOT obstruction in patients with large AML without LV hypertrophy in some situations, such as dehydration, during exercise, and inotropic treatment. Although most of patients with chronic AR have no concentric LV hypertrophy, patients with AML enlargement may suffered from LVOT obstruction in these situations.

## Study limitations

The present study has several limitations. This study was a retrospective single center study; the numbers of subgroups were relatively small and varied. A large populated and multicenter study is thus warranted. The present study did not assess the histological appearance. Since the surgeons were not able to approach the LV and MV while performing isolated aortic valvuloplasty and replacement, the MV tissues were not obtained. The severity of AR was not equally evaluated by the quantitative methods, such as the PISA method and VC width. The selections of mid-diastolic frame in the MV images were not strictly the same in all patients, although, the frame rate was enough to decrease the measurement errors in HR. In the present study, the influence of MV annulus shape due to aortic root dilatation on the severity of MR was not completely excluded; however, its influence was not significant in the analyses. We only excluded the patients with severe calcification of the MV annulus in the present study; the influence of mild MV annulus calcification on MR degree and MV Doppler parameters was not completely ruled out. Although this study included the patients with normal TEE findings as controls, they might not be truly normal because they had indication of TEE for suspected intracardiac thrombus and infective endocarditis. The selection bias possibly remained in this study. Meanwhile, the controls in the earlier studies conducted on MV geometry also had the similar prevalence of these comorbidities. Finally, the duration of AR and morphological changes were not assessed in this study. Further studies are thus required to investigate more detailed associations between the disease duration of chronic AR and MV morphological changes.

## Conclusions

3D TEE identified geometric differences in the MV apparatus between the two different AR jet directions. The present study demonstrated that an eccentric posterior AR jet enlarged the AML and restricted MV opening to a greater degree than a non-posterior jet. These results would be helpful in understanding the mechanisms of MV morphological changes in chronic AR.
